# Regional Principal Color Based Saliency Detection

**DOI:** 10.1371/journal.pone.0112475

**Published:** 2014-11-07

**Authors:** Jing Lou, Mingwu Ren, Huan Wang

**Affiliations:** School of Computer Science and Engineering, Nanjing University of Science and Technology, Nanjing, Jiangsu, China; College of Mechatronics and Automation, National University of Defense Technology, China

## Abstract

Saliency detection is widely used in many visual applications like image segmentation, object recognition and classification. In this paper, we will introduce a new method to detect salient objects in natural images. The approach is based on a regional principal color contrast modal, which incorporates low-level and medium-level visual cues. The method allows a simple computation of color features and two categories of spatial relationships to a saliency map, achieving higher F-measure rates. At the same time, we present an interpolation approach to evaluate resulting curves, and analyze parameters selection. Our method enables the effective computation of arbitrary resolution images. Experimental results on a saliency database show that our approach produces high quality saliency maps and performs favorably against ten saliency detection algorithms.

## Introduction

The reliable extraction of *ROI* (Region of Interest) allows us to detect potential regions containing objects quickly and effectively prior to goal-driven recognition processing. Since there are many potential regions to be recognized in a natural scene, we need to filter out those regions unrelated to our goal while reducing miss detection. The human visual system, tends to focus attention on the regions with high contrasts such as intensity, color and orientation [1], which makes it easier to notice those important regions in a scene. This visual attention mechanism comes from human sensitivity to contrast stimulus, and reflects a human's ability to understand natural scenes.

Saliency detection, which is related to this human visual attention mechanism, aims to judge the important parts of natural scenes automatically. It is widely used in many computer vision applications, including image segmentation [2], [3], image classification [4], [5], object recognition [6], image retrieval [7], [8], and non-photorealistic rendering [9], [10]. The results of measuring saliency values are usually represented in a saliency map, which describes how the pixels or regions stand out in the input image. In general, saliency detection methods are attributed to contrast features of image regions with their surroundings. In recent years, numerous computational models have been proposed to find the most informative region and then further analyze vision contents in a scene. Viewed from a data processing perspective [11], the commonly adopted saliency detection methods can be classified as biologically based [1] (slower, task-dependent, top-down), purely computational [12]–[16] (fast, pre-attentive, bottom-up), or a combination of these characteristics [17].

Inspired by the early primate visual system and selective attention [18], Itti *et al.* [1] compute visual features by center-surround operations, and achieve contrasts using a Difference of Gaussians (DoG) approach. In contrast, pure computational methods do not rely on biological vision. For instance, Ma and Zhang [12] use a fuzzy growth model to extend saliency maps generated based on local contrast analysis. Achanta *et al.* [13] evaluate feature distances to determine salient regions using luminance and color. Hou and Zhang [14] extract the spectral residual in spectral domain to construct the saliency maps. Li *et al.* [15] find Hou's method uses only phase information and only works in certain cases (e.g., detecting small salient regions in uncluttered scenes), then perform the convolution of the image amplitude spectrum with a low-pass Gaussian kernel of an appropriate scale to detect image saliency. Riche *et al.* [16] also propose a bottom-up visual saliency model “RARE”, which models both local contrast and global rarity using a sequential features extraction, and then fuses cross-scale rarity quantization into a single final saliency map. Furthermore, Harel *et al.* [17] use Itti's method to create feature maps and introduce Markov chains to compute saliency values. This model incorporates ideas that are based partly on biological vision principles and partly on pure computations.

In this paper, we focus on the bottom-up saliency detection method, which is mainly classified into local low-level considerations and global considerations [19]. Local low-level methods aim to continuously obtain human attention shift, and compare image regions to their local surroundings [1], [17]. These methods highlight the intersections by differences against a small neighborhood, but neglect the frequency and amount in which features may occur in cluttered backgrounds. In contrast, global contrast based methods [10], [11] evaluate saliency in the entire image. This category of methods considers global contrast differences and spatial relationships, but leads to expensive computations (e.g., measuring saliency at each pixel). Thus, by combining local saliency with global considerations, the salient objects of interest can be assigned a higher degree of importance [19], [20].

**Figure 1:**
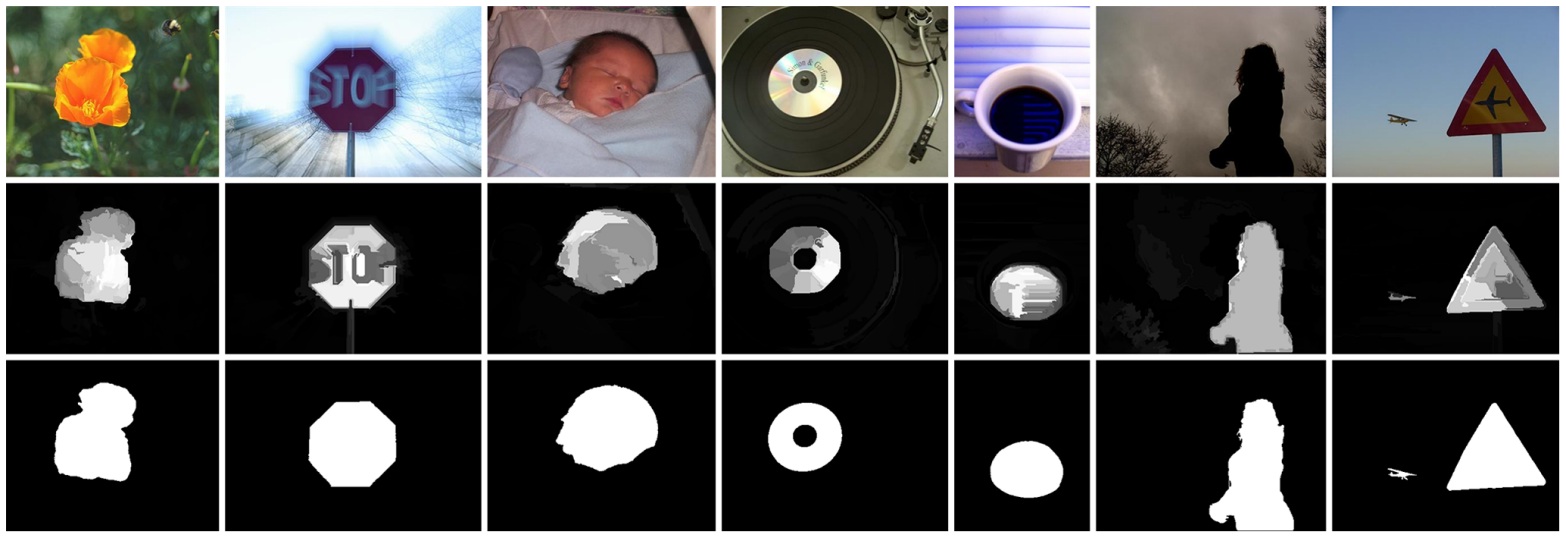
Saliency maps vs. ground truth. Given several original images [20] (**top**), our saliency detection method is used to generate saliency maps by measuring regional principal color contrasts (**middle**), which are comparable to manually labeled ground truth [11] (**bottom**).

Mainly inspired by Cheng's method [10], an automatic saliency detection method via regional principal color contrast is proposed in this paper. We exploit low-level color cues to detect saliency on the same scale as input image, and incorporate ideas that are based partly on local models and partly on global ones. Our goal, is to identify the salient regions that correspond to the manually annotated ground truth. In contrast to the methods which measure saliency with contrasting pixel-pairs, our method is more efficient by measuring region-pairs. Fig. 1 shows some saliency maps generated by our method.

In the following sections we introduce and illustrate our saliency detection algorithm. In Section *Materials and Methods*, regional principal color based saliency detection is presented. Starting from reducing the number of pixel colors to be further contrasted, we use a quantized image to build its color histogram, and obtain global color saliency based on pixel color statistics. Then we segment the quantized image into regions, and represent the saliency of each region as its principal color's saliency. Finally, we measure two categories of spatial relationships to produce full resolution saliency maps. We also introduce the details of how to select experimental parameters, and propose an assessment approach in Section *Discussion*. To evaluate the performance of our method, in Section *Results*, we compare our method with [1], [10]–[14], [17], [19], [21] and human-labeled results [11]. The results indicate that our method is an effective and reliable computational model for saliency detection.

## Materials and Methods

In this section, we present an effective regional principal color model using low-level and medium-level cues. We first present a method for detecting color saliency based on global considerations. Next, a local model is proposed based on a segmentation algorithm [22] with regional saliency represented by the saliency of the regional principal color. Finally, spatial relationships are exploited to generate a visual saliency map for the input image.

### Global Color Saliency

**Figure 2:**
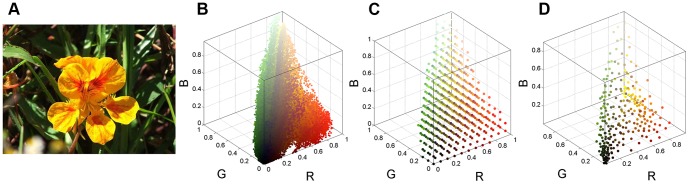
Color space distribution and quantization. (**A**) Input image [20]. (**B**) Original color distribution of **A** in the RGB color space. (**C**) Color distribution of uniform quantization. (**D**) Color distribution of minimum variance quantization.

Color contrast based methods evaluate color differences to define color saliency. As a result, the procedure obviously requires a number of comparison computations. In the 24 bits RGB full-color model (the total number of colors *N* = 256^3^), directly measuring color differences for each pair of colors takes *N*(*N*-1)/2 times. In fact, the computational complexity of contrasts in a natural image can be greatly reduced based on the following three reasons: (1) the colors in a scene occupy only a small portion of the full-color space. (2) due to the great variety of colors in a natural image, the extremely rare colors are not typically considered as salient, and often neglected. For example, in Fig. 2A, there are, in total, 62,743 different colors in the RGB space, but 75.2% of them occur only once. Although all pixels of these colors occupy approximately 39.32% of the total number of image pixels, we still can discard them to avoid voluminous color comparisons. (3) in the full-color space, human vision cannot distinguish subtle difference between two similar colors.

To reduce the number of colors in an input image, the normal treatment is image quantization. Cheng *et al.* [10] uniformly quantize each color channel of RGB model to 12 different values, which reduce the number of colors to 12^3^ = 1728. Uniform quantization subdivides the color cube into equal-sized smaller cubes, and maps the pixels within each smaller cube to the pixel color at the center of the cube. Although the uniform quantization scheme is straightforward, it does not consider the non-uniform color distribution of the input image and which results in a highly inefficient splitting of the color space [23]. By contrast, minimum variance quantization is proposed by Heckbert [24], which separates the color cube into several boxes of different sizes based on color distribution in the image, and then uses the average color in each box to create the new reduced colormap.

Let *c_x,y_* be the color value of pixel at (*x*, *y*) in the original image, *q*(*c_x,y_*) be the color value in the quantized image, and 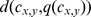 denotes the difference between corresponding color values. Then, the difference between the original and quantized images, i.e., the total quantization error, can be measured by 
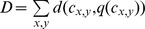
, and the optimal quantizer for a given image is defined as the one which minimizes *D*. In [24], Heckbert uses a simple color distance squared in the RGB space as the color metric. After constructing an initial colormap that assigns to each color an approximately equal number of pixels in the original image, an iterative procedure is proposed to minimize *D* between the original pixels and colors assigned from the colormap. As a result, minimum variance quantization allocates more of the colormap entries to colors that appear frequently, and fewer ones that appear infrequently. Thus, for a given number of colors in the input image, minimum variance quantization gets higher accuracy of the resulting colors.

Saliency detection tends to search for rare or infrequent features in a given image [15]. For example, Fig. 2A has many dark colors (e.g., blacks and dark greens, see Fig. 2B), which are not typically considered as salient in the image. By contrast, although yellows occur less than the dark colors (i.e., the yellows in Fig. 2A are less frequent), the flower is obviously dominant in the image. With consideration to non-uniform color distribution, minimum variance quantization allocates fewer yellows and more dark colors in the output colormap. This results in the retention of the same color rarity as the input image. Thus, in this work, we directly quantize the 24-bit RGB input to 8-bit output using minimum variance quantization, that is, we reduce the number of colors to 256 (more comparisons are discussed in Subsection *Color Quantization*). Figs. 2C and 2D illustrate the RGB color distribution of uniform quantization and minimum variance quantization of Fig. 2A, respectively. As expected, we see in Fig. 2D that there are fewer yellows and more entries to dark colors allocated in the output colormap.

**Figure 3:**
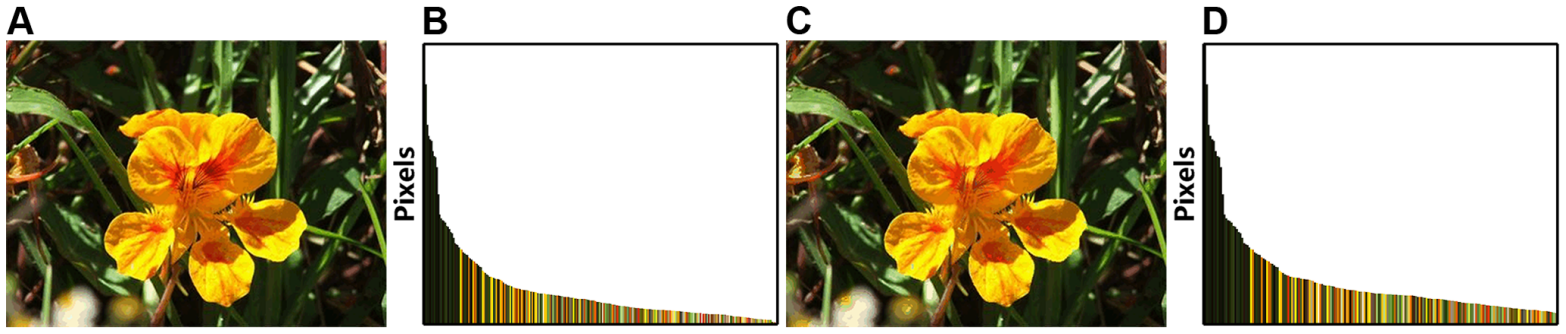
Replacement for low frequent colors with minimum variance quantization. (**A**) Minimum variance quantized Fig. 2 **A**. (**B**) Color histogram of the image in **A**. (**C**) Full resolution output image resulting from the retained high frequent colors. (**D**) Color histogram of **C**.

After quantizing the input image to 256 colors, we compute its color histogram by counting the numbers of each color in the RGB color space, and sort the color elements in descending order, so that in the output color histogram, higher frequently occurring colors contain more pixels. Considering the reason (2) given above, we abandon those infrequently occurring colors, and only choose high frequent ones. For the obtained color histogram, we accumulate the number of color pixels in descending order until retained color pixels occupy more than 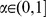 of the total number of image pixels (the selection of the best *α* is discussed in Section *Discussion*). Fig. 3A is the 8 bits (or 256 possible colors) output of the image in Fig. 2A, obtained using minimum variance quantization just discussed. Fig. 3B shows the corresponding color histogram of Fig. 3A, obtained by sorting the number of color pixels in descending order.

Then, for the remaining colors which occupy less than 1-*α* of the image pixels, we replace them respectively by the most similar colors in the color histogram. Instead of the RGB color model, we directly transform the retained colors from RGB to L^*^a^*^b^*^, and measure color differences in the L^*^a^*^b^*^ color space [10], [21], which is designed to approximate human vision. The difference between two colors 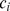 and 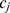 is defined as:
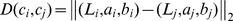
(1)where 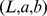 is the value of color *c* in the L^*^a^*^b^*^ color space, and 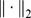 represents the *L*_2_ norm of color difference. By replacing the infrequently appearing colors in Fig. 3A, the number of colors is further reduced to 199 (see in Fig. 3D). The visual result after quantization and replacement is shown in Fig. 3C, which retains similar visualization as in Figs. 2A and 3A.

As no prior knowledge regarding the size and location of the salient object is provided, the same color in an input image can be considered to have the same saliency with global contrast differences. Thus, the saliency value of a retained high frequent color 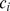 can be obtained as [10]:
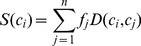
(2)where *n* is the number of retained colors, and *f_j_* is the number of pixels occupied by color 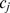. That is, the saliency value with respect to color 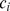 is computed as color contrast and corresponding number of pixels in other colors. Then, as in Cheng's method, we replace 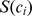 by the saliency values of *m* most similar colors in the L^*^a^*^b^*^ color space as follows (also see [10]):
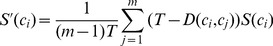
(3)


Here, the parameter *n* is as defined for Eq. (2), 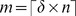 is the number of colors which are most similar to color 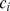, and 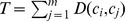 is the sum of color contrasts between 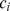 and the other colors. The parameter 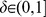 is used to control the number of most similar colors to 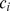, and so that the saliency value of 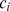 will be smoothed by all the other colors if 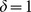. We also discuss in Section *Discussion* details of how to select the best 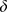.

**Figure 4:**
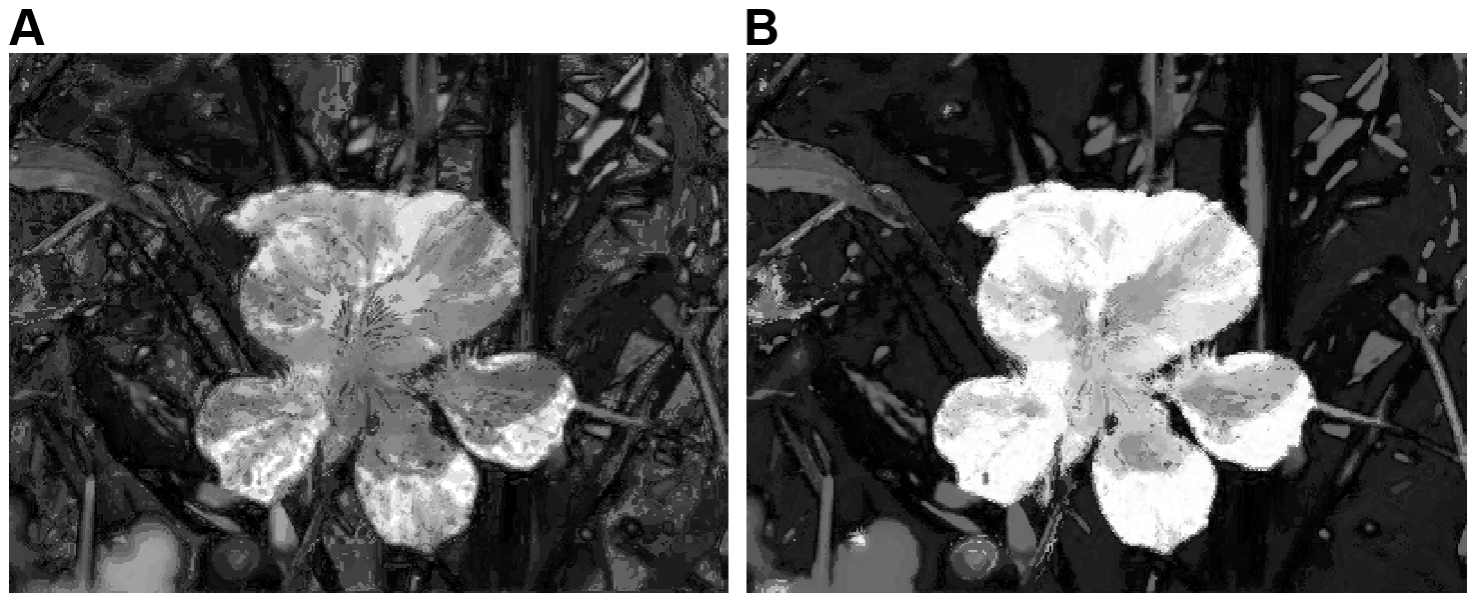
Saliency map generated by global color contrast. (**A**) Global color saliency. (**B**) Color space smoothing.

As seen in Fig. 4, we represent each color of Fig. 3C by its saliency value to generate the global saliency map (Fig. 4A), and further obtain the smoothed output in the L^*^a^*^b^*^ color space [10] (see in Fig. 4B). For the purposes of visual demonstration, all the saliency values of two figures are normalized to the range [0, 1]. Note that in Fig. 4B, the flower is assigned to higher importance after color space smoothing.

### Regional Principal Color Saliency

Compared to low-level cues, medium-level cues contain more structural information for subsequent analysis. In a natural scene, human vision tends to pay more attention to regions rather than to pixels. Region contrasts, on the other hand, are based on human subjective preferences regarding the main colors and the sizes of different regions. In addition to color contrast by statistical histogram, we introduce regional principal color for region contrast in this section.

In region contrast based on principal color, we first segment the quantized input image into regions using the graph-based image (superpixel) segmentation method [22]. In recent years, superpixels, which segment an image into several small regions of uniform color or texture [25], are widely used as a prior step in many computer vision tasks such as objectness [25] and saliency detection. The key property of superpixel segmentation is based on the idea of preserving object boundaries, that is, all pixels in a superpixel belong to the same object [26]. Due to similar colors and structural information contained in a superpixel, the pixels of a salient object can be clustered more efficiently and stably than the original pixels themselves. Li *et al.* [27] use a linear combination of the single-image saliency map (SISM) and the multi-image saliency map (MISM) to detect co-saliency. In MISM, the authors produce a pyramid of images with decreasing resolutions and decompose each into superpixels. Then, two categories of regional features are extracted to measure the similarity between co-multilayer graphs. To detect regional saliency based on sparse histogram comparisons, Cheng *et al.* [10] segment the input image into regions using [22], which is also used in [28] to detect salient objects with different scales. Xie *et al.* [29] propose an image clustering method at the superpixel level using sparse representation and apply it to compute the prior distribution of saliency. Furthermore, methods of fusing the saliency of multi-level segmentations are also adopted in [30], [31].

**Figure 5:**
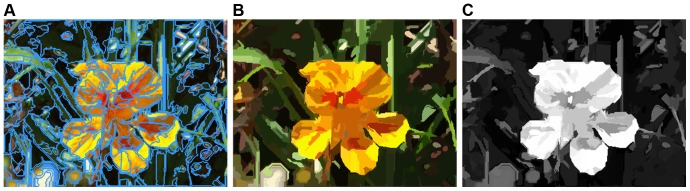
Regional principal color contrast. (**A**) Regional boundaries of using the graph-based segmentation method. (**B**) Each region represented by its principal color. (**C**) Saliency map obtained with the saliency values of regional principal colors.

In this paper, we directly use the C++ implementation provided by [22]. The primary concern of our method is the results of the superpixels clustering, where we wish to obtain color homogeneity on a minor scale. For the segmentation parameters used in our experiments, the width of the Gaussian filter is set to 0.5, scale parameter and minimum region size (pixels) are all set to 50. Fig. 5A shows the segmented result using the above parameters. There are a total of 456 regions, which are all labeled with their boundaries.

Using the segmentation procedure just described, although all colors in a single region have a high level of homogeneity, there are some other colors that also in the same region. As mentioned earlier in this section [see reason (2)], if these colors whose pixels occupy an extremely low ratio of the small local region, humans commonly ignore them, and focus only on the more frequent colors. Based on this property, we choose the most frequently occurring color (which in this paper we call the principal color) of each region respectively, and then replace the colors in each region by it, as illustrated in Fig. 5B. Note that although this procedure introduces artifacts, the flower is still salient in the image. Moreover, when ignoring some infrequently occurring colors, the number of colors in Fig. 5B is further reduced from 199 to 102, which will facilitate subsequent color contrast computations. Finally, the saliency value of region 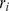, denoted 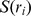, is obtained by the saliency value of the principal color 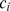 [i.e., 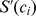 in Eq. (3)]. The image shown in Fig. 5C is the saliency map generated by principal color saliency (normalized to the range [0, 1]).

### Spatial Relationships

Generally, spatial relationships play an important role in measuring saliency in a visual space. Human visual selective mechanisms dynamically scan a scene, and shift attention to the different locations based on the center-surround principle [20], [32]. That is, the focus of attention is shifted to one salient location, and subsequently jumps from it to the next most salient location, which is spatially close to the former [1]. Furthermore, Tatler [33] and Judd *et al.* [34] find that observers tend to look more frequently around the center of the scene than around the periphery. Although there is no direct evidence that central fixation tendencies arise from central biases in natural scenes [33], the scene center is commonly used to extract global scene features for initial context modeling [35]. The concept of center-bias, that is, the salient object is usually located at the center of an image, is usually realized as Gaussian distribution and widely used in [30], [31], [36], [37].

Considering spatial distances influence on regional saliency values, we use two categories of distance metrics in our method as follows:

(1) Spatial distance between two regions

Let 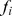 and 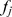 be the number of pixels in regions 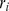 and 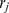, and let 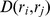 be the Euclidean distance between the center points of two regions (all of the distances are normalized to the range [0, 1]). Then, for any region 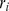, the saliency value 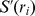 is obtained by summing spatially weighted principal color contrasts:
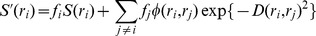
(4)where
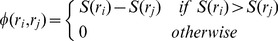
(5)


In Eq. (4), the central coordinate 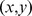 of a region is defined as the average value of *x*- and *y*-coordinates of pixels in it, and we use an exponential function to control the spatial distances so that those far-away regions would influence less on the saliency value of region 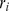. The factor 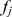 in Eq. (4) also accounts for the number of pixels in region 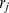, that is, bigger regions contribute more to the saliency value of region 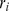 than smaller ones. In contrast to [10] which measures saliency with color contrasts between pixels in regions [evaluating the weighted saliency value for each region takes 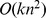 time, *k* is the number of regions and *n* denotes the number of high frequent colors], we retain the self-saliency of region 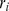, and efficiently compute region contrasts only by exploiting the principal color of each region [i.e., computing the saliency value of region 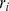 using Eq. (4) takes 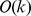 time]. Moreover, the weighting coefficient 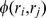 depends on the difference between the saliency values of the two regions [see Eq. (5)]. For those non-salient regions, we do not wish the saliency values of them to be indirectly increased by other sufficiently salient regions. In this case, the difference value remains unchanged when the saliency value of region 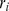 is larger than 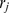, otherwise 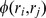 is set to zero.

**Figure 6:**
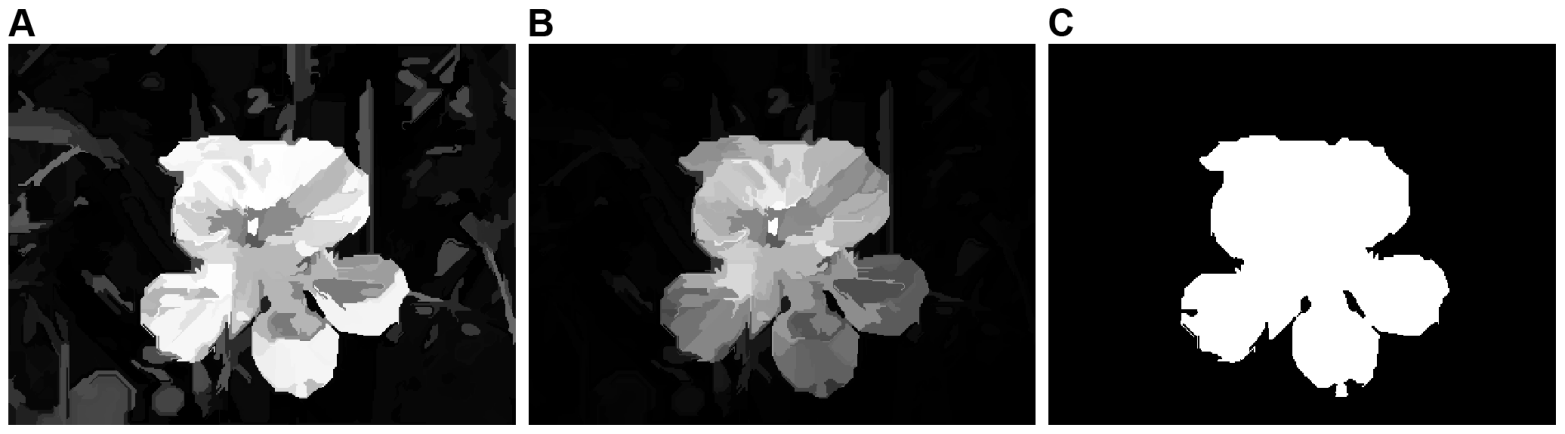
Saliency map with measuring two categories of spatial relationships. (**A**) Between two regions. (**B**) Between regional center and image center. (**C**) Binary segmented result simply obtained by thresholding **B** with an adaptive threshold.

Fig. 6A is the spatial weighted result of the image in Fig. 5C, obtained using spatial relationships across regions just discussed. Note the non-salient patch in the lower right-hand corner of Fig. 6A, in which the saliency is further remarkably restrained. In contrast to Fig. 5C, our method decreases the saliency of background regions, and improves the visual contrasts between salient and non-salient regions.

(2) Spatial distance between regional center and image center

Let *C* represent the coordinate 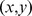 of the center point of Fig. 6A, and let 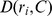 denote the Euclidean distance between the center of region 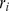 and the image center. Like the first category of distance metric just discussed, all of the distances are also normalized to the range [0, 1]. A simple approach to measure this category of distance metric is direct controlling spatial weighting, where we compute an estimate, 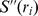, of the saliency value of region 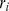 simply by dividing 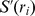 by the exponential function of the weighted 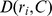, as follows:
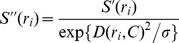
(6)where, as noted at the beginning of this subsection, the parameter 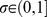 is the strength of the response of the spatial weighting used to compute 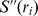. This indicates that, for a fixed value of 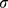, the regions which are further away from the image center, will be assigned smaller saliency values than those immediate neighbors of the image center. However, the sufficiently salient regions are still more salient with an appropriate 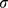, even if Eq. (6) makes regions around the periphery have lower saliency. Similarly, we will discuss the selection of the best 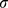 in Section *Discussion*. The image in Fig. 6B is obtained using Eq. (6) with 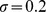. In comparing this image with Fig. 6A, we note a generally slight decrease of saliency throughout the entire image but, as expected, those non-salient regions around the image are considerably more affected.

Finally, the image shown in Fig. 6C is simply obtained by thresholding Fig. 6B with an adaptive threshold (see Section *Results* for a discussion). Comparing this result with the manually labeled ground truth, we get a high quality object, and see that it is a reasonable representation of what we would consider to be salient in Fig. 2A.

## Results

**Figure 7:**
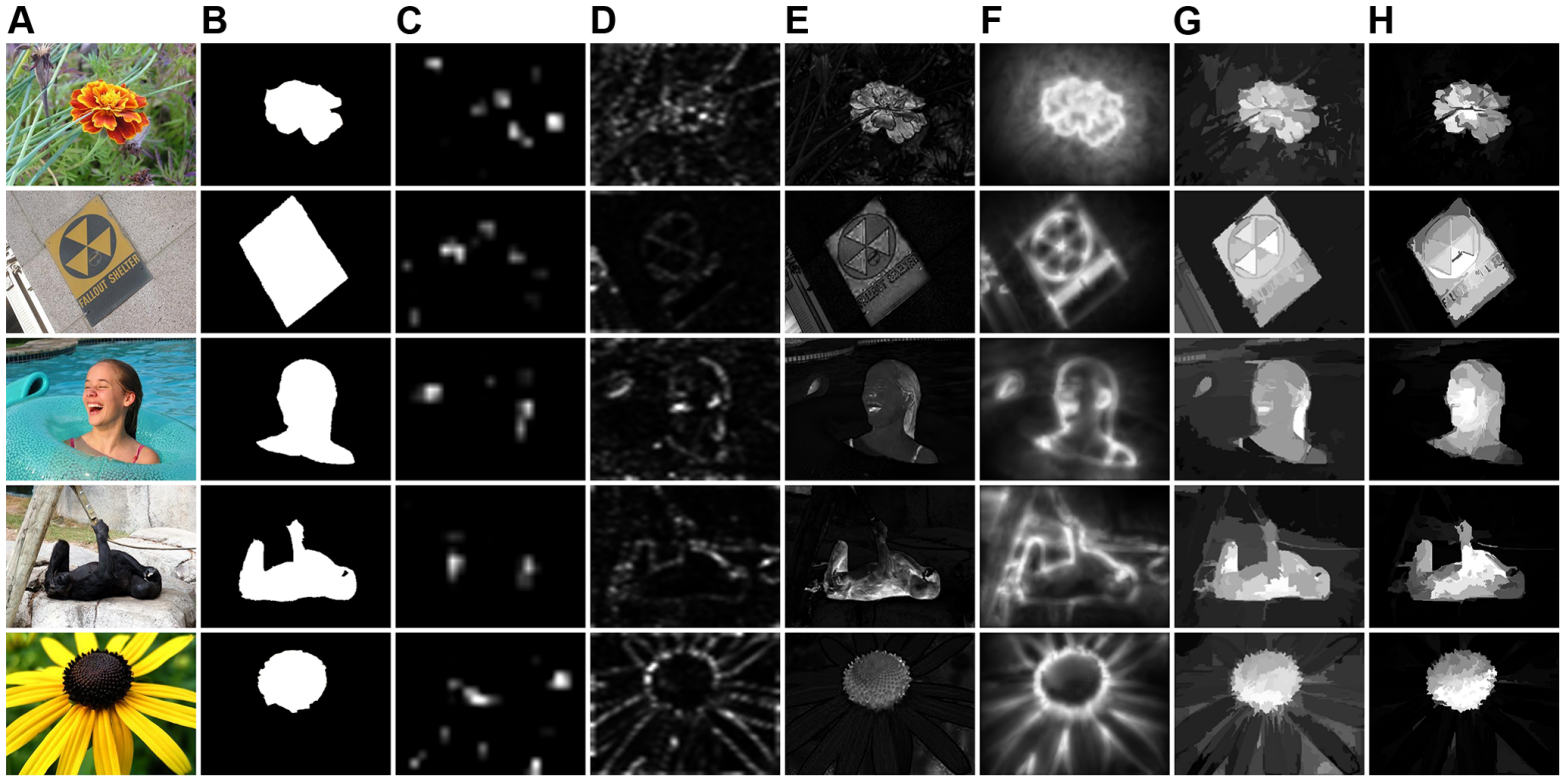
Visual results of our method compared with ground truth and other methods on dataset MSRA-1000. (**A**) Original images [20]. (**B**) Ground truth [11]. (**C**) IT [1]. (**D**) SR [14]. (**E**) FT [11]. (**F**) CA [19]. (**G**) RC [10]. (**H**) Ours.

We present empirical evaluation and analysis of the proposed method against ten saliency detection methods on the MSRA-1000 salient object database [20], with the manually labeled ground truth provided by Achanta *et al.* [11], including IT [1], AC [13], GB [17], MZ [12], SR [14], FT[11], CA [19], LC [21], HC [10], RC [10]. In our experiments, for all ten methods mentioned above, we directly use the saliency maps provided by Cheng *et al.* [10]. Fig. 7 shows some visual sample results of our method compared with others, where higher pixels indicate higher saliency. Our method can effectively handle complex foreground and background.

In order to objectively evaluate the accuracy of our method for salient object detection, following the settings in [11], three comparison measures are used in our experiments. For an input image, let *A* denote the manually labeled ground truth, and let *B* represent the binary segmented result of our saliency map, we compute precision and recall value (denoted as *P* and *R*, respectively), as follows [38]:
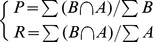
(7)


Since *A* and *B* are all binary, we simply use the logical *AND* operation (i.e., 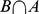) to obtain the intersection of these two images (1-valued), and use the expression 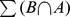 to denote the number of pixels in the image regions where *A* and *B* overlap. Moreover, to acquire both high precision and high recall, we evaluate F-measure [11] (represented by *F*) as:
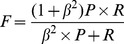
(8)here, we also use *β* ^2^ = 0.3 to weigh precision more than recall.

For a given saliency map, with the saliency values in the range [0, 255], we performed two experiments to compare the segmentation results with ground truth. In the first experiment, we binarize the saliency map with 256 fixed thresholds. In the second experiment, the saliency map is segmented by adaptive thresholding.

### (1) Segmentation by fixed thresholding

**Figure 8:**
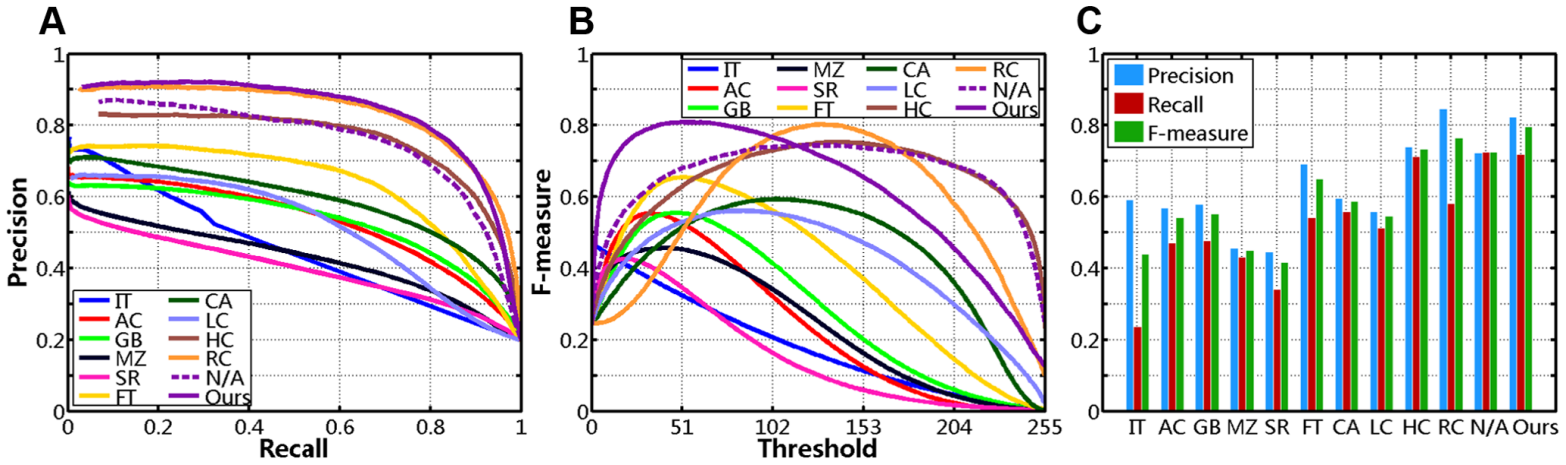
Quantitative comparison on dataset MSRA-1000 (N/A represents no center-bias). (**A**) Precision-Recall curves. (**B**) F-measure curves. (**C**) Precision-Recall bars.

For every image from MSRA-1000, we vary the threshold *T_f_* from 0 to 255 sequentially, and obtain 256 binary segmentation results. Then, we compute the average precision, recall, F-measure of 1000 images at each possible threshold, and plot precision-recall curves (as shown in Fig. 8A) and F-measure curves (see Fig. 8B). Generally, the accuracy of the saliency maps can be observed in the plots of precision-recall curves, and we wish to achieve higher precision in the entire recall range. However, because each method produces different precision and recall values at the same threshold, this comparison method is rough and subjective. In quantitative evaluation, we use an interpolation approach to measure the precision-recall curves objectively.

We first generate a linearly spaced vector of *n* points (with *n* = 1000 in this case) in the entire recall range [*R_min_*, *R_max_*]. Then, we compute 1000 interpolated values of precision using linear interpolation, and obtain the average value of them (represented by *P_s_*). The first row of Table 1 compares the average interpolated precision taken by each method. In contrast, we produce the highest *P_s_*, which means that our approach achieves higher precision in most recall range (see in Fig. 8A).

**Table 1:** Numeric comparison on data set MSRA-1000 (%, N/A represents without center-bias).

	IT [1]	AC [13]	GB [17]	MZ [12]	SR [14]	FT [11]	CA [19]	LC [21]	HC [10]	RC [10]	N/A	Ours
*P_s_*	45.3	53.1	53.1	42.8	39.9	63.2	58.9	51.4	75.4	83.2	75.0	**83.6**
*P*	59.2	56.8	58.0	45.6	44.5	69.2	59.6	55.8	74.0	**84.6**	72.4	82.4
*R*	23.7	47.1	47.6	43.2	34.1	54.2	55.9	51.2	71.4	58.1	**72.6**	71.9
*F*	44.0	54.2	55.2	45.0	41.6	65.1	58.7	54.7	73.4	76.5	72.5	**79.7**

### (2) Segmentation by adaptive thresholding

Most saliency detectors exploit saliency maps in salient objects segmentation. For instance, Ma and Zhang [12] use fuzzy growing on their saliency maps to find rectangular salient regions. Achanta *et al.* [13] retain only the regions whose average saliency is greater than a constant threshold, and further improve this method with an adaptive threshold [11]. Cheng *et al.* [10] iteratively apply GrabCut [39] to refine the segmentation results initially obtained by thresholding the saliency maps.

Although more sophisticated approaches can be used to obtain more accurate segmentation results, simple thresholding reflects the essence of saliency maps making salient objects stand out. In other words, the accuracy of saliency detection methods depends entirely on how well the saliency maps can be obtained. Inspired by [11], [38], in our experiments, we directly determine the adaptive threshold (*T_a_*) as twice the average value of all saliency regions:
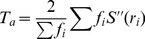
(9)where, 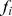 and 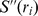 in this equation are as defined in Eqs. (4) and (6). For a saliency map, any regional saliency value greater than the threshold *T_a_* is set to 1 (white), and all others are set to 0 (black).

With *T_a_* as set in Eq. (9), we obtain binary segmented results of salient objects from each saliency detection method. Average values of precision, recall and F-measure are also obtained over 1000 images from the MSRA-1000 database, as mentioned earlier in this section. The comparisons are shown in Fig. 8C, and the last three rows of Table 1. Among all the methods, our method achieves the best accuracy with higher recall (71.89%), and the best F-measure (79.74%). The RC model [10] achieves higher precision scores but their recall value is much lower than our algorithm. Note that, our method computes regional contrasts by exploiting only 102 principle colors in Fig. 5A, and uses the simplest thresholding in salient object segmentation.

**Figure 9:**
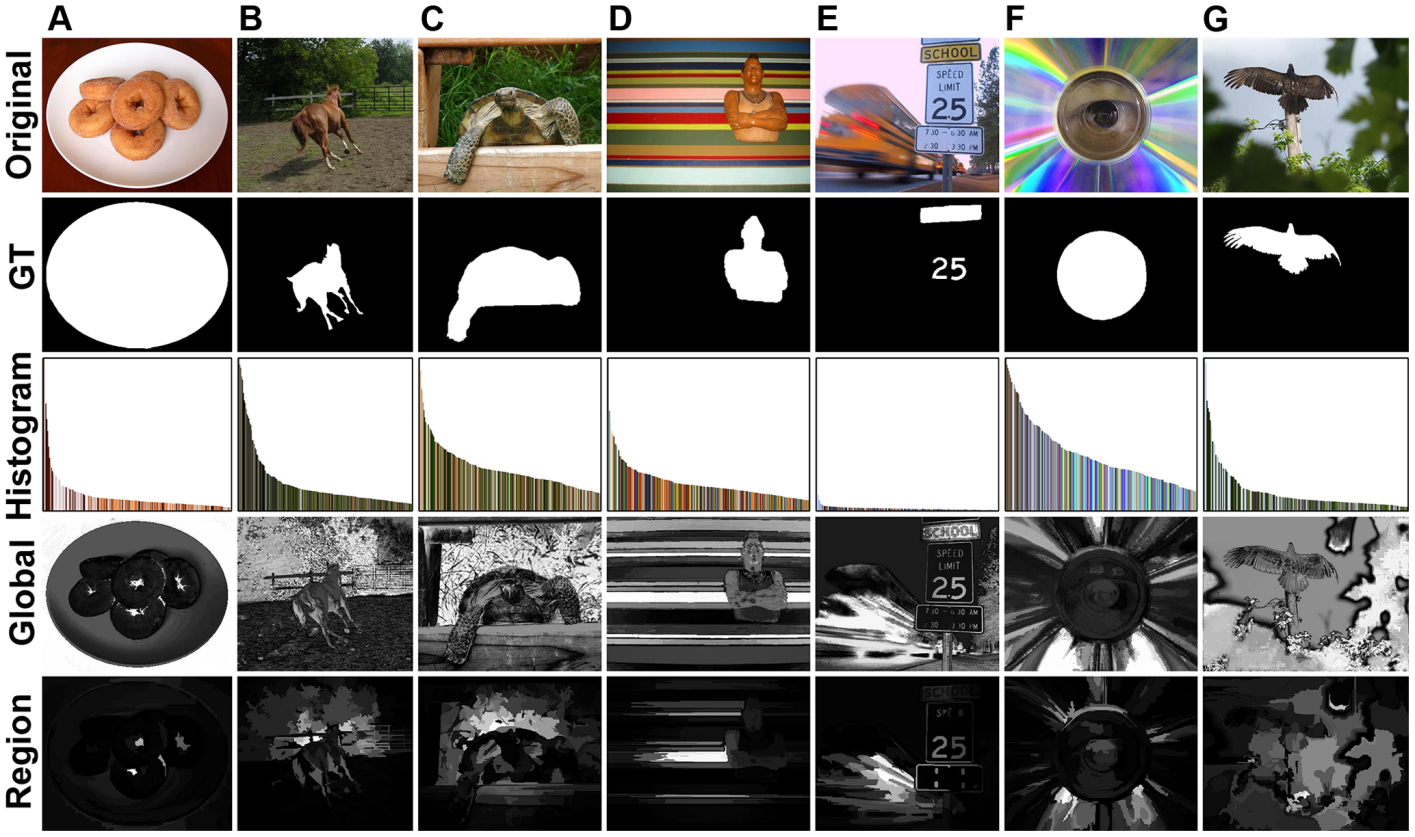
Hard image cases of our method in detecting salient regions. (Top to bottom) Original images [20], ground truth (GT) [11], color histogram similar to Fig. 3 **D**, global color contrast, and regional principal color based saliency detection.

Although our method performs well in experimental results, it does fail in some cases. Here, we respectively segment the 1000 saliency maps by adaptive thresholding *T_a_* in Eq. (9) as described earlier, and sort the F-measure values of binary results in descending order. From the last ten images, we collect several of the incorrect saliency detection results in Fig. 9. The third row of Fig. 9 is the color histogram similar to Fig. 3D, the forth row is the saliency maps generated based on global color contrast (see Subsection *Global Color Saliency*) and the last row is the final results. As an improvement over global contrast based method, regional principal color based saliency detection method produces incorrect results while the former can incur wrong responses. Why does global color contrast based saliency detection method fail in these cases?

Saliency detection amounts to searching for unusual features in a given image [16], which means that those salient regions are expected to be rare or infrequent when compared with other regions [15]. In this paper, our method employs only low-level color features to detect saliency, it cannot satisfactorily deal with salient parts with similar colors as the non-salient regions. For example, in Figs. 9B and 9C, color features of human-labeled ground truth are similar to other parts in the scenes (ground and wood). In certain cases (e.g., both Fig. 9F and Fig. 9G), manually labeled parts consist of high frequent colors, which are not usually considered as salient regions. Similarly, in Fig. 9A, the salient objects occupy the large parts of the image, but our method locates the centers of the doughnuts with infrequent dark colors. Furthermore, our bottom-up model is not task-dependent, it totally fails to highlight the salient regions in Figs. 9D and 9E.

There are two ways to alleviate these issues: one is to introduce more complex features (intensity, orientations, etc.) [1] or biological vision principles [17], the other is to adopt a multi-scale strategy (as used in [1], [15], [16]) or hierarchical model [31]. The first method introduces prior knowledge or task information not included in our bottom-up saliency model, and predicts human eyes fixations using attention trajectories to locate the regions of visual attraction. The second method considers that cue maps could be quite different in different scales, and multiple layers would contain both small regions and large-scale structures. By fusing multi-layer saliency maps, this category of approaches is able to highlight both small and large salient regions.

## Discussion

### Color Quantization

As described earlier in Subsection *Global Color Saliency*, in contrast to Cheng's method [10], we use minimum variance quantization to reduce the number of colors in the input images. To compare the accuracy of two kinds of quantization methods in our saliency detection model, we perform two groups of experiments using the optimal combination of 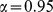, 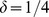 and 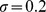 as discussed in the following subsection. In these experiments, we follow the same procedure, except that we make the changes in color quantization. In the first group of experiments, we uniformly quantized each color channel of RGB model to 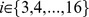 different values, so the resulting colors are varied from 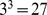 to 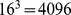. In the second group of experiments, we directly quantize the 24-bit RGB input to *i*-bit output (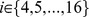) using minimum variance quantization. Moreover, we add an additional 1728 into the second group of experiments.

**Figure 10:**
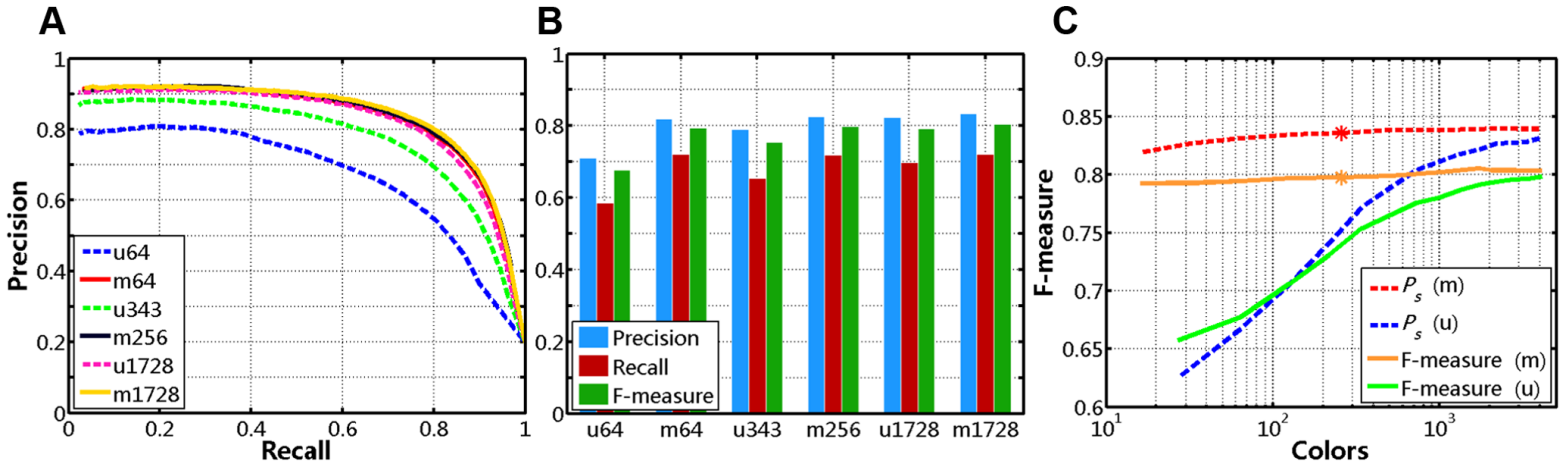
Uniform quantization vs. minimum variance quantization. (**A**) Precision-Recall curves. (**B**) Precision-Recall bars. (**C**) F-measure curves.

Then, for every image from MSRA-1000, we follow the same procedure mentioned in Section *Results*. With 256 fixed thresholds, we compute the average precision, recall, F-measure of 1000 binary results segmented by fixed thresholding, then the average interpolated precision (*P_s_*) is obtained in the entire recall range (as shown in the first row of Tables 2 and 3). Several of the precision-recall curves are plotted in Fig. 10A (uniform quantization denoted as *u*, and minimum variance quantization denoted as *m*). Furthermore, with *T_a_* in Eq. (9), all saliency maps are segmented by adaptive thresholding, and we obtain the average values of precision (*P*), recall (*R*), F-measure (*F*) over the whole 1000 images (see the last three rows of Tables 2 and 3). Several of the precision-recall bars and F-measure curves are also plotted in Figs. 10B and 10C, respectively.

**Table 2:** Numeric comparison for various colors used in uniform quantization (%).

Colors	27	64	125	216	343	512	729	1000	1331	1728	2197	2744	3375	4096
*P_s_*	62.6	66.7	70.5	74.1	77.2	78.9	80.4	81.1	81.8	82.1	82.7	82.7	82.9	83.2
*P*	68.4	71.1	74.0	76.6	79.0	80.0	81.1	81.4	81.9	82.4	82.6	82.9	82.9	83.1
*R*	58.2	58.5	61.1	63.3	65.4	66.8	67.7	68.6	69.4	69.9	70.2	70.3	70.5	70.7
*F*	65.7	67.7	70.6	73.1	75.4	76.5	77.6	78.0	78.7	79.1	79.4	79.6	79.6	79.8

**Table 3:** Numeric comparison for various colors in minimum variance quantization (%).

Colors	16	32	64	128	256	512	1024	1728	2048	4096
*P_s_*	81.88	82.67	83.13	83.45	83.59	83.86	83.85	83.89	83.97	83.94
*P*	81.99	81.85	81.98	82.31	82.44	82.66	83.07	83.47	83.29	83.30
*R*	71.36	71.76	72.04	71.95	71.89	71.93	72.05	72.06	71.99	71.89
*F*	79.26	79.28	79.45	79.66	79.74	79.91	80.24	80.53	80.38	80.36

First, as can be seen in Table 2 and Fig. 10C, the precision, recall and F-measure values of uniform quantization increase significantly as the number of colors increases. By contrast, these measures obtained using minimum variance quantization increase slowly (see Table 3). Second, two kinds of quantization methods have similar performance while employing adequate colors (e.g., 4096), but resulting tremendous color comparisons. Finally, comparing Table 2 with Table 3, we can see that minimum variance quantization is superior to uniform quantization, while providing the same number of colors. Thus, in consideration of achieving high accuracy and simultaneously reducing computational complexity in our method, we quantize the input images to 8-bit output using minimum variance quantization.

### Parameter Selection

As mentioned in previous sections, so far there are three undetermined parameters to be used in our approach:

(1) The value of *α* is the ratio of the pixels of high frequent colors to the total number of image pixels (see Subsection *Global Color Saliency*). As *α* increases, more colors in the quantized image will be retained, but increasing color contrast computations.

(2) The value of 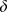 in Eq. (3) is the ratio of the most similar colors (to 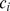) to the total number of colors in saliency smoothing. To reduce quantization artifacts, more similar regional principal colors to 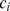 will be used to smooth the saliency value of region 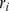, as 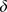 increases.

(3) The value of 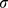 in Eq. (6) is the spatial weighting to measure the effect of distance between region 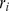 and image center. As 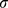 decreases, the resulting saliency maps would be more center-biased. That is, the salient regions mainly concentrate on image center, with farther regions being assigned smaller saliency values.

The three parameters *α*, 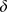, and 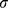 are therefore selected as follows. In order to acquire the optimal selection, we consider the various combinations associated with particular values, and perform two experiments using different comparison measures as mentioned earlier in Section *Results*. Our ultimate goal is to obtain both the best average interpolated precision value (*P_s_*) in salient object segmentation by fixed thresholding, and the best F-measure (*F*) in segmentation by adaptive thresholding.

**Figure 11:**
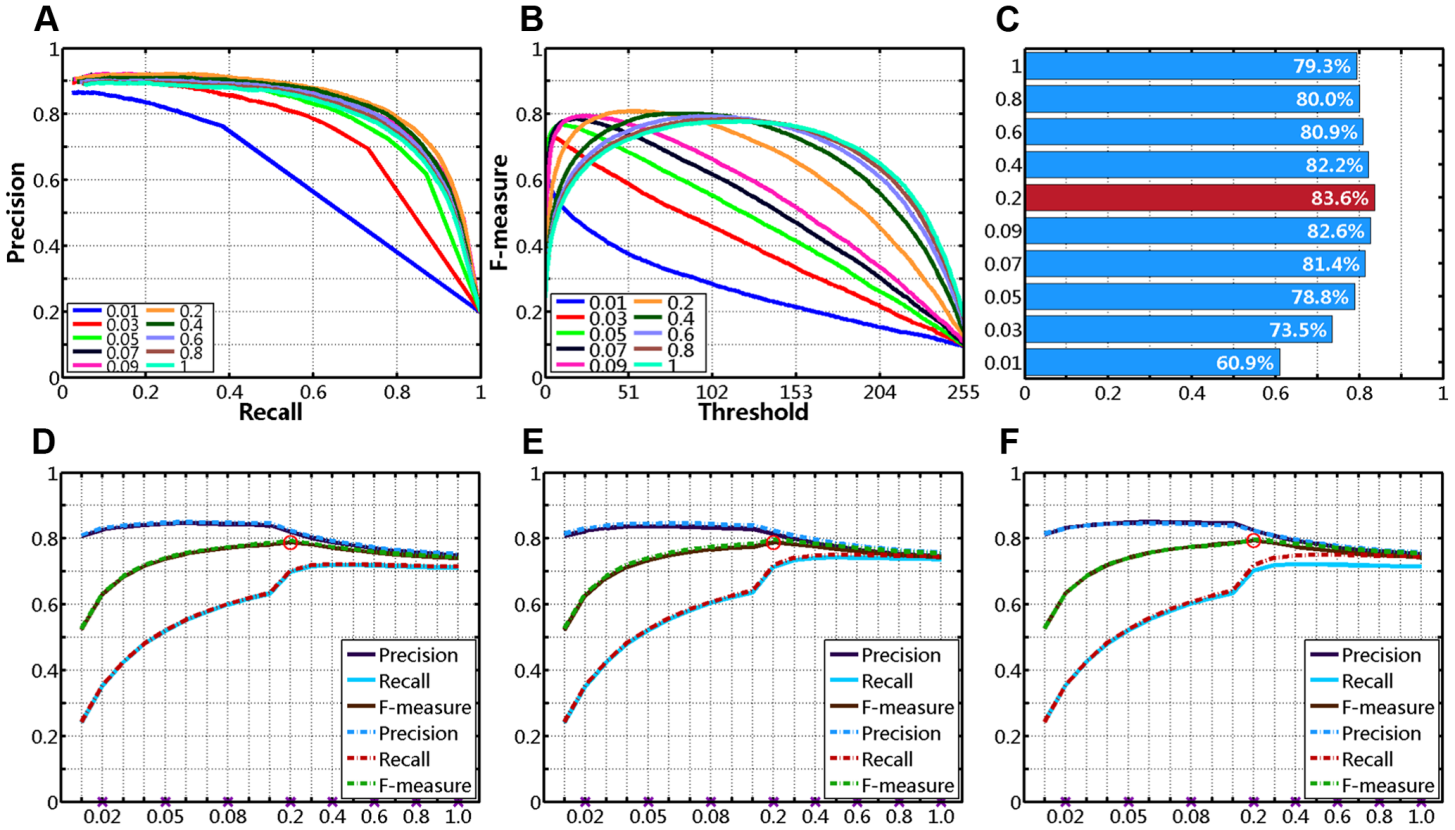
Quantitative comparison for various combinations of parameters. (**A**)–(**C**) Varying σ from 0.01 to 1 with *α* = 0.95 and *δ* = 1/4: (**A**) Precision-Recall curves. (**B**) F-measure curves. (**C**) *P_s_* Bars. (**D**)–(**F**) Plots of precision, recall, and F-measure for various values of σ: (**D**) *α* = 0.9, *δ* = 1/16 vs. *α* = 0.95, *δ* = 1/16. (**E**) *α* = 0.9, *δ* = 1/4 vs. *α* = 0.95, *δ* = 1/4. (**F**) *α* = 0.95, *δ* = 1/16 vs. *α* = 0.95, *δ* = 1/4.

In our experiments, *α* is set to 0.9 or 0.95, and 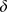 is set to 1/16 or 1/4 (partly referred to the work of [10]). In terms of the parameter 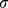 of Eq. (6), we set it to a value in two possible closed intervals: [0.01, 0.09] with a step size of 0.01, or [0.1, 1] with a step size of 0.1 (a total of 19 values; only ten are chosen for demonstration purposes in this paper). For an arbitrary set of parameter combination, in the first experiment, we measure the average interpolated precision values (*P_s_*) in the entire recall range. The first row of Fig. 11 shows the precision-recall curves, F-measure curves and *P_s_* bars, for ten different values of 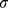 respectively, with 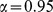 and 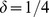. And as shown in Fig. 11C and Table 4, we obtain the best *P_s_* (83.6%) with 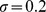 (*y*-axis) in this case.

**Table 4:** Numeric comparison for various combinations of parameters (%).

σ	*α* = 0.9, *δ* = 1/16				*α* = 0.9, *δ* = 1/4				*α* = 0.95, *δ* = 1/16				*α* = 0.95, *δ* = 1/4			
	*P_s_*	*P*	*R*	*F*	*P_s_*	*P*	*R*	*F*	*P_s_*	*P*	*R*	*F*	*P_s_*	*P*	*R*	*F*
1.0	76.4	74.9	71.2	74.0	77.7	74.6	73.7	74.4	77.3	75.2	71.5	74.3	79.3	75.8	74.5	75.5
0.8	77.3	75.7	71.5	74.7	78.3	75.3	74.0	75.0	78.1	76.1	71.8	75.0	80.0	76.6	74.8	76.1
0.6	78.4	77.0	71.8	75.7	79.2	76.7	74.2	76.1	79.3	77.3	72.1	76.0	80.9	77.9	75.1	77.2
0.4	80.2	78.9	72.0	77.2	80.5	78.6	74.1	77.5	80.9	79.2	72.2	77.5	82.2	79.7	74.9	78.6
0.2	**82.4**	82.1	70.0	**78.9**	**82.2**	81.3	71.4	**78.8**	**83.1**	82.5	70.3	**79.3**	**83.6**	82.4	71.9	**79.7**
0.09	81.8	84.3	61.8	77.8	81.4	83.1	62.3	77.2	82.3	84.7	62.0	78.1	82.6	84.1	62.7	78.0
0.07	80.6	84.6	57.7	76.4	80.2	83.6	58.2	75.9	81.0	84.9	58.0	76.7	81.4	84.6	58.7	76.8
0.05	78.1	84.5	51.9	73.8	77.8	83.7	52.1	73.4	78.4	84.8	52.2	74.1	78.8	84.5	52.5	74.1
0.03	72.7	83.6	42.5	68.4	72.7	83.2	42.5	68.1	73.0	84.0	42.7	68.7	73.5	83.9	42.7	68.7
0.01	60.4	80.8	24.2	52.5	60.4	80.8	24.1	52.4	60.6	81.1	24.4	52.7	60.9	81.5	24.4	52.9

In the second experiment, we segment the saliency maps generated by our method using an adaptive threshold *T_a_* as set in Eq. (9), and mainly measure the average F-measure values over 1000 input images. Table 4 shows the numeric comparison of the average interpolated precision (*P_s_*), precision (*P*), recall (*R*) and F-measure (*F*) for various combinations of *α*, 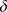 and 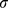. Note that we always achieve the best *P_s_* and the best *F* with 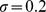, regardless of the combinations of *α* and 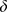. On the other hand, in all combinations discussed here, we obtain almost the best metrics with *α* = 0.95 and 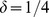. Thus, we use fixed parameters of 0.95, 1/4, 0.2 for *α*, 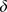, and 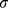 in our method, respectively, for all the images from MSRA-1000.

Furthermore, in visual comparison, we plot precision, recall, F-measure for various combinations of *α*, 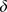, and 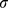 by adaptive thresholding, as shown in the second row of Fig. 11. As illustrated in Fig. 11D, the image in it shows three sets of curves of *α* = 0.9, 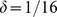 (solid lines) vs. *α* = 0.95, 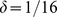 (dash-dot lines) with varying 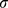 from 0.01 to 1 (*x*-axis). Similarly, two plots of comparison curves for other combinations of *α*, 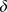, and 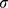
are shown in Figs. 11E and 11F. First, Figs. 11D–11F show that we can achieve higher precision, recall, F-measure with bigger *α* at the same 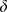, and higher recall with bigger 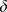 at the same *α*. On the other hand, the precision values increase slowly as 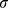 increases, and reach the peak when 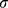 is approximately equal to 0.1. The F-measure curves have similar increasing trends, but will decrease slowly when 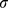 is approximately bigger than 0.2.

### Center-bias Factor

As discussed in Subsection *Spatial Relationships*, we employ the spatial distance between regional and image centers [see Eq. (6)], and hence introduce the center-bias effect in our saliency detection model. In [15], Li *et al.* consider that the center-bias factor affects the equality of measuring ROC directly, and then calibrating this post-processing in order to make a fair comparison. To eliminate the influence of center-bias, we discard the second spatial relationship, and compute the average interpolated precision (*P_s_*) using fixed thresholding, the average precision (*P*), recall (*R*), F-measure (*F*) using adaptive thresholding. The experimental results are shown in Fig. 8 and Table 1, in which N/A represents no center-bias.

As can be seen in Table 1, although N/A still achieves the best recall (72.6%), its performance is close to HC [10] but lower than RC [10]. There are three factors leading to the experimental results. First, as mentioned in Subsection *Regional Principal Color Saliency*, we represent regional saliency value as only the saliency value of its principal color, and hence reduce precision of regional contrasts while decreasing computation complexity. Second, to avoid the saliency values of non-salient regions to be indirectly increased by other sufficiently salient regions, we set the weighting coefficient 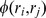 to zero [see Eq. (5)], resulting in partly suppressed sizes and saliency values of the salient regions. Finally, it should be admitted that the dataset itself has a certain built-in bias [40]. Since the performance of our method is significantly improved while introducing center-bias factor, the salient regions on MSRA-1000 are mostly located at the image centers.

## Conclusions

In this paper, we propose a principal color method via regional contrasts and spatial relationships to detect salient regions in natural scenes. We use low-level color features to build color histograms based on minimum variance quantization, and compute global color saliency in the quantized image. Then, we segment the quantized image into regions, and define the saliency for each region using the saliency value of the regional principal color. Based on two categories of spatial relationships at the region level, the spatial weighting schemes are subsequently introduced. In addition, we present an interpolation approach in order to quantitatively evaluate precision-recall curves, and discuss color quantization, parameters selection and center-bias factor. We evaluate the proposed method on a data set of 1,000 typical images with manually labeled ground truth. Experimental results demonstrate the effectiveness of our method. In contrast to measuring regional saliency differences with pixel-pairs, our method incorporates low-level and medium-level visual cues, and computes saliency with region-pairs while remaining simple, which consequently, can be easily applied in *ROI* extraction, image segmentation, and potential object detection for natural scenes.

The ultimate goal of our research is to develop a pedestrian detection system using multi-spectral image sensors. In the future, first, we plan to employ more effective features to deal with hard image cases. In this paper, we only use low-level color features to detect saliency, which results in failure when the salient parts and non-salient regions have similar colors. Since the regional principal color based detection method is effective, it is desirable to introduce other principle features extracted from image cues to handle the cluttered images. Second, in this paper, only one saliency map is generated as the final result. Due to the changeful scales of pedestrian in the images, the method of fusing multi-layer saliency maps is able to highlight both small and large pedestrian regions. Third, because pedestrians are partly salient in the infrared images, we plan to introduce prior knowledge of pedestrian structures, and iteratively employ multiple center-biased salient regions in the initial salience map to improve the detection results. Finally, the proposed method is not real-time and needs several seconds to operate the algorithm in a MATLAB implementation. However, we have noted that a decrease of about 0.5% of the performances can make our algorithm twice as fast. Our future work consists in the optimization of the proposed model so that it works in real-time.
